# Untargeted ultra-high-resolution mass spectrometry metabolomic profiling of blood serum in bladder cancer

**DOI:** 10.1038/s41598-022-19576-9

**Published:** 2022-09-07

**Authors:** Joanna Nizioł, Krzysztof Ossoliński, Aneta Płaza-Altamer, Artur Kołodziej, Anna Ossolińska, Tadeusz Ossoliński, Tomasz Ruman

**Affiliations:** 1 Faculty of Chemistry, Rzeszów University of Technology, 6 Powstańców Warszawy Ave., 35-959 Rzeszow, Poland; 2 Department of Urology, John Paul II Hospital, Grunwaldzka 4 St., 36-100 Kolbuszowa, Poland; 3 Doctoral School of Engineering and Technical Sciences at the Rzeszów University of Technology, 8 Powstańców Warszawy Ave., 35-959 Rzeszow, Poland

**Keywords:** Chemistry, Analytical chemistry, Cancer, Urological cancer, Biomarkers

## Abstract

Bladder cancer (BC) is a common urological cancer of high mortality and recurrence rates. Currently, cystoscopy is performed as standard examination for the diagnosis and subsequent monitoring for recurrence of the patients. Frequent expensive and invasive procedures may deterrent patients from regular follow-up screening, therefore it is important to look for new non-invasive methods to aid in the detection of recurrent and/or primary BC. In this study, ultra-high-performance liquid chromatography coupled with ultra-high-resolution mass spectrometry was employed for non-targeted metabolomic profiling of 200 human serum samples to identify biochemical signatures that differentiate BC from non-cancer controls (NCs). Univariate and multivariate statistical analyses with external validation revealed twenty-seven metabolites that differentiate between BC patients from NCs. Abundances of these metabolites displayed statistically significant differences in two independent training and validation sets. Twenty-three serum metabolites were also found to be distinguishing between low- and high-grade of BC patients and controls. Thirty-seven serum metabolites were found to differentiate between different stages of BC. The results suggest that measurement of serum metabolites may provide more facile and less invasive diagnostic methodology for detection of bladder cancer and recurrent disease management.

## Introduction

Bladder cancer (BC) is the second most frequently diagnosed cancer of the urinary tract after prostate cancer in the world. In 2020, this disease affected over 473,000 individuals worldwide and was responsible for 212 536 deaths^1^. According to TNM Classification of Malignant Tumors system proposed by American Joint Committee on Cancer (AJCC), bladder cancer can be classified according to whether the tumor infiltrates into or out of the muscular tissue as muscle-invasive bladder cancer (MIBC) and non-muscle-invasive bladder cancer (NMIBC) respectively^2^. NMIBC is the most common type of BC and includes noninvasive papillary carcinomas (pathologic stage Ta), submucosal invasive tumors (T1) and carcinoma in situ (CIS). MIBC includes tumor which extends into the muscle (stage T2), into the perivisceral fat layer (stage T3) or nearby organs (stage T4). Statistically, in case of 80% of patients tumor do not spread outside of the bladder wall. BC can also be classified by histology as low-grade (LG) tumor that rarely spread from their primary site, and high-grade ones (HG) that are more aggressive and invasive^3^.

Generally, the first treatment for early BC is a trans urethral resection of bladder tumor (TURBT) sometimes followed by intravesical instillation of mitomycin or *Bacillus* Calmette-Guerin (BCG) therapy. On the other hand, standard treatment for MIBC is a radical cystectomy with pelvic lymph-node dissection. This is combined with neoadjuvant or adjuvant cisplatin based chemotherapy^4^. Despite such aggressive type of treatment, the survival rate of bladder cancer patients is low. Thus, it is essential to combine local and systemic therapies to improve outcomes. High-grade tumors are usually detected by cytology with high specificity and selectivity, but in the case of low-grade tumors, their determination is very difficult.

Metabolomic instrumental analysis is powerful family of tools mainly often used for study of biofluids. Small molecules levels in biofluids such as serum reflects the current state of the organism allowing for identification and characterization of potential disease biomarkers. The number of metabolomics studies in the diagnosis and understanding of many diseases is rapidly growing in recent years^5^. Numerous analytical methods have been used to better understand the metabolic changes occurring in living systems and especially cancer phenotypic changes. However, two analytical platforms including nuclear magnetic resonance (NMR) spectroscopy^6^ and mass spectrometry (MS) often coupled with liquid chromatography (LC)^7^ allow to achieve the most comprehensive screening of cancer metabolomes. MS in comparison to NMR, allows the detection of much broader range of compounds with much higher sensitivity, resolution, and precision using very small amount of sample^8^. Over the past fifteen years, metabolomic analytical methods have been used extensively to investigate BC and to identify potential biomarkers of this cancer in urine, serum, and tissues^9,10^. Compared to urine, serum metabolomics is less prone to be affected by dilution factor. Serum is also more readily available than tissue and procedure less invasive^11^. Despite the advantages of examining the metabolomes of human sera, there are only a few studies on serum metabolomics focused on BC biomarker discovery. So far, most studies related to the analysis of serum of patients with bladder cancer have been carried out using NMR^12–14^ or mass spectrometry coupled with liquid^7,15–19^ and gas chromatography (GC) ^3,20,21^. The first such study of serum from BC patients with LC–MS is from 2012, when Lin et al.^22^ analyzed serum profiles of BC with LC–MS, and revealed five potential biomarkers for diagnosis of different types of genitourinary cancer. Five years later Tan et al. (Tan 2017) analyzed serum metabolites of 120 BC patients and 52 healthy persons using ultrahigh performance liquid chromatography (UHPLC) coupled with quadrupole time-of-flight (Q-TOF) mass spectrometry in conjunction with univariate and multivariate statistical analyses. They selected and validated 3 differential metabolites including inosine, acetyl-*N*-formyl-5-methoxykynurenamine and phosphatidylserine, PS(*O*-18:0/0:0) that could discriminate HG and LG BC patients and also LG BC and healthy controls. In the same year, Sahu et al. applied GC and LC–MS to identify metabolite associated with urothelial carcinoma in 72 patients and 7 patients without urothelial neoplasia^17^. Their research indicated potential metabolic pathways altered in NMIBC and MIBC BC. In 2019, Vantaku et al. presented serum targeted metabolomic analysis based on LC–MS to investigated to investigate the molecular differences in BC patients from different parts of the world. The study included two independent cohorts of 54 European Americans and 18 African Americans patients and corresponding healthy controls^16^. In the same year, Amara et al.^15^ applied LC–MS for targeted analysis of serum metabolites of 67 BC smokers and 53 post-operative BC patients and 152 healthy controls. Their research showed that serum analysis before and after tumor resection can reveal progressive and significant changes of concentration of selected metabolites. In 2021, Troisi et al. applied LC–MS to profile serum metabolites of 64 patients with BC, 74 patients with RCC, and 141 healthy controls. They used different ensemble machine learning models in order to identify metabolites that differentiate cancer patients from controls and allow to classify the tumor in terms of its stage and grade (Troisi 2021).

In this work we report the first results of untargeted analysis of human sera with ultra-high-resolution mass spectrometry coupled to ultra-high-performance liquid chromatography. This study employed the large number of patients—100 cancer patients and 100 controls. Untargeted analysis was focused on serum metabolic changes generated by bladder cancer but also stratifying the disease by stage and grade. Our study reveals potential BC biomarkers for early detection, screening and differential diagnosis.

## Materials and methods

All chemicals were of analytical reagent grade. Deionized water (18 MΩ cm) was produced locally. LC–MS-grade methanol was bought from Sigma Aldrich (St. Louis, MO, USA).

### Instrumentation

Instrumental configuration consisted of a Bruker Elute UHPLC system operated by Hystar 3.3 software and a ultra-high-resolution mass spectrometer Bruker Impact II (60,000+ resolution version; Bruker Daltonik GmbH) ESI QTOF-MS equipped with Data Analysis 4.2 (Bruker Daltonik GmbH), and Metaboscape (2021b). A Waters UPLC column ACQUITY BEH (C18 silica, 1.7 μm particles, 50 × 2.1 mm) with compatible column guard was used for all analyses. Two mobile phases were: A = Water with 0.1% formic acid, B = acetonitrile with 0.1% formic acid (v/v). Samples in autosampler were thermostated at 4 °C temperature. Volume of 5 μL of extract was loaded on the column at a flow rate of 200 μL min^−1^, using 4% B. B percentage was changed with time as follows: 0 min—1%, 0.56 min—1% B, 4.72 min—99%, 5.56 min—99%, 5.6 min—1%, 9.45 min—1%. Solvent flow was 450 μL min^−1^. Column was thermostated at 40 °C temperature. Internal calibration on 10 mM sodium formate (water: isopropanol 1:1 v/v) ions was performed automatically in Metaboscape with the use of syringe pump at an infusion flow rate of 0.12 mL h^−1^, using a high precision calibration (HPC) mode. Analyses in positive autoMSMS mode were carried out using the following parameters: *m/z* 50–1200; capillary voltage: 4.5 kV; nebulizer: 2.7 bar; dry gas: 12 L min^−1^; drying gas temperature: 220 °C; hexapole voltage: 50 Vpp; funnel 1: 200 Vpp; funnel 2: 200 Vpp; pre-pulse storage time: 5 μs; transfer time: 60 μs. Collision-Induced Dissociation (CID) was used with following settings: absolute threshold (per 100 sum): 200 cts; absolute threshold 88 cts; active exclusion 3 spectra; release after 0.3 min, isolation mass: for *m/z* = 100, width was 3, for 500 width was 4, for 1000 was 6 and for 1300 was 8); collision energy value was 30 eV. MS frequency was 20 Hz and MSMS from 5 to 30. The untargeted annotations were performed in Metaboscape (ver. 2021b) with a criterion of mass deviation (Δ*m/z*) under 2 ppm and mSigma value under 15 as the maximum acceptable deviation of the mass of the compound and the isotopic pattern respectively. For identification and molecular formula generation, exact mass of parent ions was matched with < 3 ppm error and mSigma value < 50 in most cases. All the molecular formulas were obtained using the Smart Formula tool and the C, H, N, O, P, S, Cl, Br, I and F elements. MSMS spectra was automatically matched against MSMS libraries: Bruker HMDB 2.0 library, MassBank of North America (MoNA)^23^ library and NIST ver. 2020 MSMS library^24^. The quality control (QC) sample were prepared from 100 different serum extracts and were measured every ten samples throughout the analytical run to provide a set of data from which method stability and repeatability can be assessed. All measurements were made in technical triplicates.

### Collection of human blood samples

Serum samples were collected from one hundred bladder cancer patients (average age 73, Caucasian race) at John Paul II Hospital in Kolbuszowa (Poland). Control serum samples were collected from healthy volunteers after medical examination focused on detection of urinary cancers. All the patients underwent transurethral resection of bladder tumor (TURBT) following detailed clinical questioning and laboratory testing. The study was approved by local Bioethics Committee at the University of Rzeszow (Poland, permission no. 2018/04/10) and performed in accordance with relevant guidelines and regulations. All patients involved in the study were informed about the purpose of this research and planned procedures, and signed an informed consent form. Just over half of the patients (n = 54) had low-grade bladder cancer and papillary urothelial neoplasm of low malignant potential (PUNLMP) (n = 3), while the remaining patient group exhibited high-grade disease (n = 41). In two cases, both high- and low-grade neoplasms were detected. The majority of these patients (n = 69) displayed noninvasive papillary carcinomas (pathologic stage Ta, pTa) stage disease, nineteen had submucosal invasive tumors (pathologic stage T1, pT1) stage and twelve patients had muscle invasive bladder cancer (pathologic stage T2, pT2). The average age for diagnosed patients with BC was 74 ± 10 years while in NCs group the average age was 64 ± 12. The entire NCs group consists of patients admitted to the urology department for surgical treatment of benign urological conditions (urolithiasis, benign prostate hyperplasia, testicular hydrocele, varicocele, phimosis, ureteropelvic junction stenosis, urinary incontinence, urethral stricture). Each of these patients has had performed at least an abdominal ultrasound to rule out neoplasms (patients with urolithiasis usually also had a computed tomography (CT) scan) and a basic bundle of lab tests required for urological surgery that rule out inflammation. Patients were selected according to a similar age range. After familiarizing patients with the research program, patients from the control group gave written consent to donate residual serum for study (no additional blood was drawn for the purpose of this study, except that taken before urological surgery). The clinical characteristics of the patients are presented in supplementary information 1, table S1. Approximately 2.6 ml of blood was drawn from each participant. Samples were centrifuged at 3000 rpm for 10 min at room temperature. The serum was then separated and kept at − 60 °C until further use.

### Sample preparation

Polar metabolites were extracted from serum samples as described in our recent publication (Nizioł, Ossoliński, et al. 2021). In brief, deep frozen blood plasma samples (300 µL) were thawed on ice to 4 °C before use. Samples were then centrifuged at 12,000×*g* for 5 min also at 4 °C temperature. Volume of 300 µL of serum was pipetted into sterile 2.0 mL Eppendorf tubes and room-temperature acetone (900 µL) was added and vial vortexed for 1 min. Resulting suspension was incubated at room temperature for 20 min followed by 30 min at − 20 °C. Tubes was then centrifuged at 6000×*g* for 5 min at 4 °C temperature to sediment serum precipitated proteins and phospholipids and then clarified supernatant A (800 µL) was transferred to a new 2 ml microcentrifuge tube. Volume of 500 µL of a 3:1 acetone/H_2_O solution was added to the pellet and vortexed vigorously until the pellet was resuspended, this tube was then centrifuged at 12,000×*g* for 10 min at 4 °C to sediment serum precipitated proteins again. Resulting supernatant B was then combined with supernatant A. Volume of 260 µL of combined supernatants were vacuum dried in speedvac-type concentrator and dissolved in 400 µL of methanol, vortexed and centrifuged (12,000×*g* for 5 min at 4 °C). Supernatant volume of 100 µL was transferred into HPLC vial insert of 130 µL capacity and inserted into Elute autosampler.

### Multivariate statistical analysis

All metabolite datasets exported from Metaboscape v.2021b were analyzed using the MetaboAnalyst 5.0 online software^25^. Prior to analysis, data was log-transformed, auto-scaled and normalized by sum. Resulting metabolite profiles were then subjected to unsupervised Principal Component Analysis (PCA). The separation between the BC and control groups observed in the 2D and 3D PCA scores plot was further examined using the supervised multivariate statistical analysis such as Orthogonal Partial Least Squares Discriminant Analysis (OPLS-DA). The quality of the OPLS-DA models was assessed by the goodness of fit (R^2^Y) and the predictive ability of the models (Q^2^). VIP plots were generated to recognize metabolites most significantly responsible for groups separation. Metabolites with VIP value higher than 1.0 were considered potential biomarker candidates. To test the accuracy of the multivariate statistical models, and to rule out that the observed separation in the OPLS-DA is due to chance (p < 0.05), permutation tests were performed with 2000-fold repetition. Statistical significance of metabolite level differences was assessed with paired parametric t-test using Mann–Witney and Bonferroni correction. *P* values and false discovery rates (FDR; q-value) less than 0.05 were considered statistically significant. Receiver operating characteristic curve (ROC) analyses together with random forest modeling were commenced to evaluate the diagnostic value of all selected metabolites. The performance of the metabolites was estimated using the area under the curve (AUC), 95% confidence interval, specificity and selectivity. Only variables with an AUC value higher than 0.75 were considered to be relevant. Multivariate statistical analyses were performed independently for the training and validation datasets. Compounds differentiating between tumor and control serum samples were selected based on external validation, which uses two independent datasets (here called training and validation dataset) to validate the performance of a model^26^. The final set of potential BC biomarkers selected fulfilled all criteria in both testing and validation data sets. Chemometric tools such as 2D PCA, OPLS-DA and ROC analysis were also used to assess metabolic profile similarities and differences between different grades and stages of bladder cancer. To identify metabolic pathways impacted by bladder cancer, a metabolic pathway impact analysis was made in MetaboAnalyst 5.0 and the Kyoto Encyclopedia of Genes and Genomes (KEGG) pathway library for *Homo sapiens*^27^. Quantitative pathway enrichment analysis was conducted based on Small Molecule Pathway Database (SMPD). Each impacted pathway was classified according to statistical *p* value, Holm p (*p* value adjusted by Holm–Bonferroni method) and FDR (*p* value adjusted using False Discovery Rate), calculated from pathway topology analysis.

### Ethics approval

The study protocol was approved by local Bioethics Committee at the University of Rzeszow (Poland) (permission no. 2018/04/10).

## Results

In this study, we characterized the metabolic profiles of one-hundred patients suffering from bladder cancer, in an effort to develop serum-specific metabolic signatures for early and specific detection of bladder cancer. For this purpose, we recorded ultra-high-resolution LC–MS spectra of 200 total (100 BC and 100 control = NCs) metabolite extracts from patient and healthy control serum samples in an effort to identify potential discriminant biomarkers of bladder cancer. Datasets from the BC patients and NCs were divided into two groups, a training set, comprising 80% of all samples and a validation set, corresponding to 20% of all samples. Patient samples of a given stage of BC in the training set accounted for 80% of all samples of that stage. Serum metabolic profiling was performed independently on the two datasets. The training set was used to identify serum diagnostic markers for cancer and stage of its malignancy and, in turn, the validation set was used to independently validate the diagnostic performance of serum metabolite biomarkers.

### Distinguishing between bladder cancer and control serum samples

**Figure 1:**
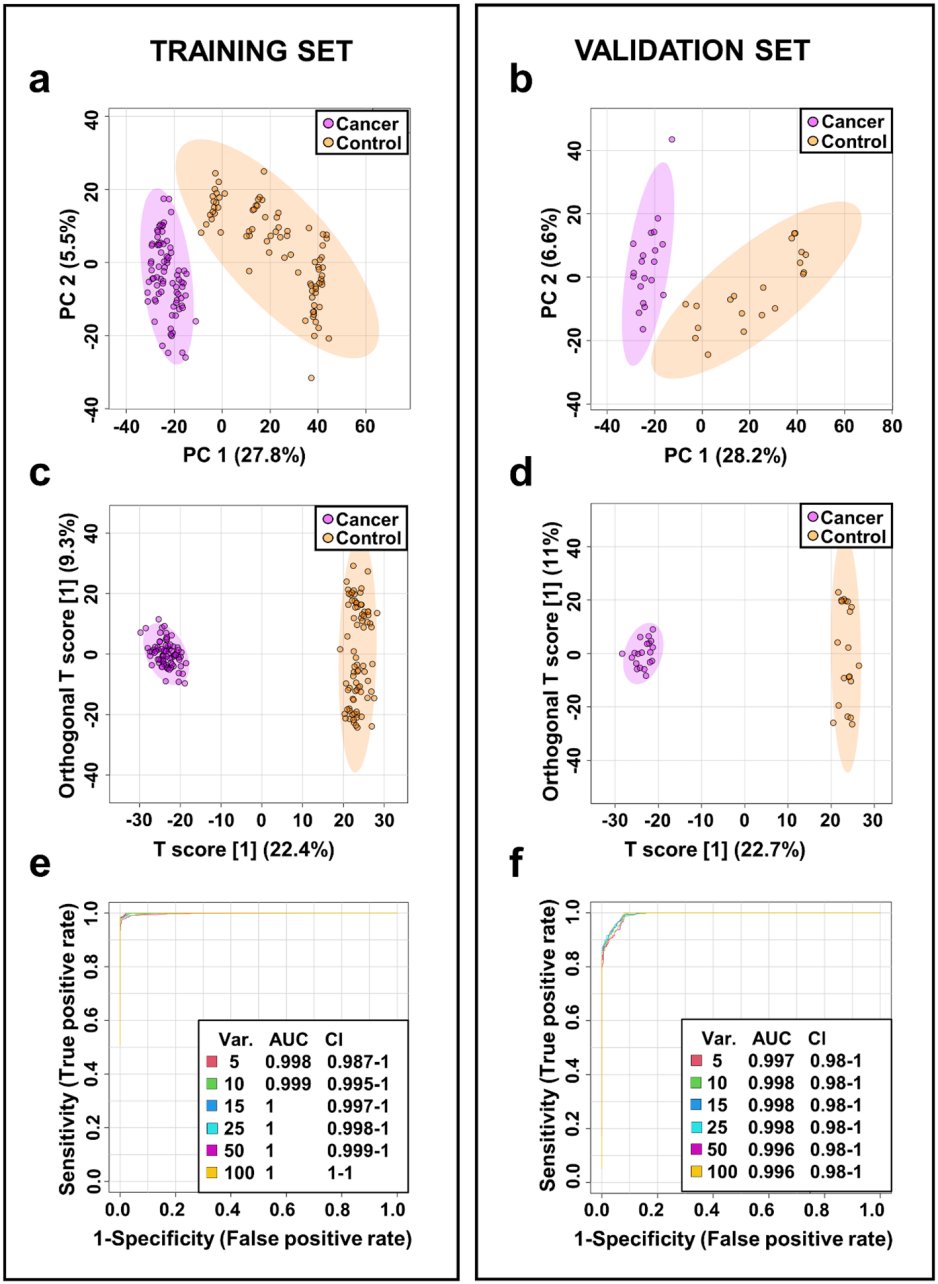
Metabolomic analysis of serum samples from BC and NCs. PCA and OPLS-DA scores plots of the tumor (violet) and control (orange) serum samples in the training set (**a**,**c**) and validation set (**b**,**d**). The receiving operator characteristic (ROC) curves in the training set (**e**) and validation set (**f**).

In total, 5498 m*/z* features were found in each serum sample in both training and validation set with applied filtration that required that software show only features that were in at least nine samples. Unsupervised 2D PCA score plots of both subsets indicated a good separation between cancer patients and controls based on distinct and characteristic metabolite profiles. The best separation of groups in the training set was obtained along principal components 1 and 2 (i.e. PC1 and PC2) which accounted for 27.8% and 5.5% respectively. Only a few outliers were detected in the central 95% of the field of view (Fig. 1a). In turn, in the validation set, the best separation between cancer and control serum samples was also observed along PC1 (28.2%) and PC2 and (6.6%) (Fig. 1b).

A supervised multivariate analysis using OPLS-DA analysis was carried out to explore the metabolic differences between the BC and NC groups. In the training set, the score plot indicated a clear separation between those two groups (Fig. 1c). Two thousand permutation tests were conducted to validate the OPLS-DA model (Fig. S1 A). Good discrimination was observed between the two groups (Q^2^ = 0.971, R^2^Y = 0.992, *p* value < 5E-04 (0/2000)), revealing substantial differences in the metabolic profiles of cancer versus control serum samples. Model overview showing high R^2^Y and Q^2^ indicating good interpretability and predictability by this OPLS-DA model (Fig. S1 B). A similar tendency to discriminate BC patients and NCs was observed in OPLS-DA model of the validation set (Fig. 1d), which was confirmed by the very good results of the permutation test (Q^2^ = 0.929, R^2^Y = 0.995, *p* value < 5E-04 (0/2000)) (Fig. S1C). Potential serum bladder cancer biomarkers were selected on the basis of the VIP plot resulting from the OPLS-DA model. By combining the VIP (> 1.0) with the results from the independent t-test (*p* value and FDR from t-test < 0.05) 1012 variables were selected in training set as differential for BC patients and NCs (Table 1, Supplementary information 2). In turn, in validation set 1052 variables were considered as significant (Table 2, Supplementary information 2). Finally, 864 common *m/z* and rt values were indicated, both in the training and validation sets. Among these features, to 121 m*/z* values were assigned to a specific chemical compound (Table 1). Next, univariate ROC analysis was separately performed on both training and validation sets to evaluate the diagnostic ability of the models. The results indicated that in the serum samples all 85 out of previously selected 121 metabolites exhibit very high area under the curve (AUC) above 0.8. As shown in Fig. 1e,f, the combination of mass features in both subsets was found to be a powerful discriminator of control versus bladder cancer serum samples (AUC > 99%). Finally, set of twenty-seven potential BC biomarkers were selected with cut-off criteria of FC > 2 and < 0.5, Δ*m/z* < 2 ppm and mSigma < 50 in both testing and validation data sets. The sensitivity and specificity of the selected 27 metabolites were also determined and all metabolites disclosed sensitivity and specificity greater than 77 and 85%, respectively (Table 1 and S1, Supplementary information 2).

**Table 1:** Differential metabolites for discrimination between BC patients and NCs (*p* value < 0.05; FDR < 0.05; VIP > 1; FC < 0.5 and > 2).

No	Name	Structure	m/z^a^	Δm/z [ppm]	RT [s]	VIP^b^	FC^c^	*p* value	FDR	AUC	Spec. [%]^d^	Sens. [%]^d^
1	Aureonitol^e,g,h^	C_13_H_18_O_2_	207.1378	− 0.7	173.2	1.82	0.20	5.00E−27	3.20E−25	0.993	96	98
2	Norcamphor^e,g,h^	C_7_H_10_O	111.0803	− 1.0	143.2	1.79	0.29	5.80E−27	3.30E−25	0.992	96	100
3	Perillyl alcohol^e,g^	C_10_H_16_O	153.1273	− 0.8	210.3	1.83	0.25	6.30E−27	3.30E−25	0.992	96	100
4	Thymol^e,f,g^	C_10_H_14_O	151.1116	− 0.8	204.2	1.78	0.39	7.30E−27	3.40E−25	0.991	94	99
5	Methyl 2-octynoate^e,g,h^	C_9_H_14_O_2_	155.1065	− 1.3	177.7	1.72	0.31	8.40E−27	3.40E−25	0.991	95	98
6	3,5,5-Trimethyl-2-cyclohexen-1-one^e,g,h^	C_9_H_14_O	139.1116	− 0.9	193.2	1.83	0.17	9.80E−27	3.50E−25	0.990	96	99
7	Alantolactone^e,g,h^	C_15_H_20_O_2_	233.1535	− 0.3	194.6	1.80	0.23	9.80E−27	3.50E−25	0.990	94	98
8	4-Heptanone^e,f,g^	C_7_H_14_O	115.1116	− 0.9	206.7	1.79	0.24	1.00E−26	3.50E−25	0.990	94	96
9	7-Epi-Jasmonic acid^e,g,h^	C_12_H_18_O_3_	211.1328	− 0.2	160.9	1.80	0.19	1.50E−26	4.10E−25	0.988	96	96
10	Dihydrojasmone^e,g,h^	C_11_H_18_O	167.1429	− 0.8	228.3	1.78	0.41	1.60E−26	4.20E−25	0.988	94	94
11	Valeric acid^e,f,g^	C_5_H_10_O_2_	103.0753	− 0.8	132.2	1.75	0.41	2.30E−26	5.10E−25	0.987	91	96
12	4,4,7a-trimethyl-3a,5,6,7-tetrahydro-3*H*-indene-1-carboxylic acid^e,g,h^	C_13_H_20_O_2_	209.1534	− 0.8	197.8	1.75	0.4	5.50E−26	9.20E−25	0.983	94	95
13	1-Acetylindole^e,g,h^	C_10_H_9_NO	160.0757	0.1	131.1	1.65	2.13	1.50E−25	2.20E−24	0.978	93	98
14	Linoleic acid^e,g^	C_18_H_32_O_2_	281.2473	− 0.8	258.6	1.52	2.55	3.50E−25	4.40E−24	0.975	94	94
15	1-Phenyl-1-pentanone^e,g,h^	C_11_H_14_O	163.1116	− 0.8	200.5	1.73	0.34	7.50E−25	8.30E−24	0.971	94	86
16	Umbelliferone^e,g,h^	C_9_H_6_O_3_	163.0389	− 0.6	182.1	1.69	0.49	1.10E−24	1.10E−23	0.970	91	90
17	Elaidic acid^e,g^	C_18_H_34_O_2_	283.2629	− 0.9	278.4	1.67	3.33	1.20E−24	1.30E−23	0.969	95	94
18	3-Ethylphenol^e,g,h^	C_8_H_10_O	123.0803	− 0.9	122.9	1.72	0.36	1.30E−24	1.30E−23	0.969	96	95
19	D-Limonene^e,g^	C_10_H_16_	137.1324	− 0.7	143.9	1.72	0.30	2.90E−24	2.60E−23	0.965	91	90
20	6-Hydroxy-4,4,7a-trimethyl-5,6,7,7a-tetrahydrobenzofuran-2(4*H*)-one^e,h^	C_11_H_16_O_3_	197.1171	− 0.8	143.2	1.60	0.41	8.60E−23	6.00E−22	0.950	89	90
21	LysoPE(P-18:0/0:0) ^e,g,h^	C_23_H_48_NO_6_P	466.3288	− 1.0	294.2	1.58	0.38	1.30E−22	8.40E−22	0.948	86	91
22	Palmitoleoyl Ethanolamide^e,g,h^	C_18_H_35_NO_2_	298.2738	− 0.9	236.5	1.41	2.08	1.50E−22	1.00E−21	0.947	90	91
23	PE(P-16:0e/0:0) ^e,g,h^	C_21_H_44_NO_6_P	438.2977	− 0.6	267.5	1.48	0.48	4.70E−20	2.30E−19	0.920	85	88
24	3-Hexanone^e,g,h^	C_6_H_12_O	211.1328	− 0.2	160.9	1.40	0.50	5.20E−18	2.10E−17	0.896	86	96
25	Epsilon-caprolactam^e,f,g,h^	C_6_H_11_NO	114.0914	− 0.2	114.5	1.16	2.35	5.70E−18	2.30E−17	0.896	85	81
26	L-Acetylcarnitine^e,f,g^	C_9_H_17_NO_4_	204.1230	− 0.3	22.9	1.37	2.36	1.50E−16	5.40E−16	0.878	85	78
27	LysoPC(18:3)^e,g,h^	C_26_H_48_NO_7_P	518.3236	− 1.0	237.9	1.25	0.48	1.30E−15	4.60E−15	0.866	86	78

^a^Experimental monoisotopic mass; ^b^VIP scores derived from OPLS-DA model; ^c^fold change between cancer and control serum calculated from the abundance mean values for each group—cancer-to-normal ratio; ^d^ROC curve analysis for individual biomarkers; ^e^the metabolites identified by high precursor mass accuracy; ^f^the metabolites identified by matching retention time; ^g^the metabolites identified by matching isotopic pattern; ^h^the metabolites identified by matching MS/MS fragment spectra; AUC: area under the curve; CI: confidence interval; FC: fold change; FDR: false discovery rate; *m/z*: mass-to-charge ratio; RT: retention time; Sens.: Sensitivity; Spec.: Specificity; VIP: variable influence on projection.

**Table 2:** Differential metabolites for discrimination between LG and HG BC patients and NCs (*p* value < 0.05; FDR < 0.05; VIP > 1; FC < 0.5 and > 2).

No	Metabolites	Formula	*m/z*^a^	RT [s]	HG versus control				LG versus control			
					VIP^b^	FC^c^	Spec. [%]^d^	Sens. [%]^d^	VIP^b^	FC^c^	Spec. [%]^d^	Sens. [%]^d^
1	Aureonitol ^e,g,h^	C_13_H_18_O_2_	207.1378	173.22	1.82	0.20	98	97	1.84	0.20	100	96
2	7-Epi-Jasmonic acid ^e,g,h^	C_12_H_18_O_3_	211.1328	160.86	1.80	0.19	96	97	1.83	0.19	96	96
3	3,5,5-Trimethyl-2-cyclohexen-1-one ^e,g,h^	C_9_H_14_O	139.1116	193.16	1.86	0.17	95	94	1.87	0.17	98	93
4	Alantolactone ^e,g,h^	C_15_H_20_O_2_	233.1535	194.63	1.80	0.25	95	94	1.84	0.22	98	93
5	Valeric acid^e,f,g^	C_5_H_10_O_2_	103.0753	132.22	1.78	0.41	98	94	1.79	0.41	95	93
6	4-Heptanone ^e,g,h^	C_7_H_14_O	115.1116	206.71	1.78	0.24	96	97	1.81	0.23	96	93
7	Methyl 2-octynoate ^e,g,h^	C_9_H_14_O_2_	155.1065	177.72	1.77	0.30	98	91	1.84	0.32	98	98
8	4,4,7a-trimethyl-3a,5,6,7-tetrahydro-3*H*-indene-1-carboxylic acid ^e,g,h^	C_13_H_20_O_2_	209.1534	197.75	1.74	0.40	98	91	1.75	0.40	96	89
9	Thymol ^e,g,h^	C_10_H_14_O	151.1116	204.15	1.74	0.39	99	94	1.76	0.39	99	93
10	Umbelliferone ^e,g,h^	C_9_H_6_O_3_	163.0389	182.05	–	–	–		1.74	0.49	94	91
11	4,7-Dimethyl-1,3-benzothiazol-2-ylamine ^e,g,h^	C_9_H_10_N_2_S	179.0638	142.04	1.66	2.00	93	88	–	–	–	–
12	D-Limonene^e,g^	C_10_H_16_	137.1324	143.88	1.66	0.30	89	94	1.70	0.31	86	93
13	LysoPE(P-18:0/0:0) ^e,g,h^	C_23_H_48_NO_6_P	466.3288	294.22	1.63	0.36	91	91	1.55	0.39	86	87
14	1-Acetylindole ^e,g,h^	C_10_H_9_NO	160.0757	131.14	1.58	2.14	93	94	1.61	2.13	98	93
15	6-Hydroxy-4,4,7a-trimethyl-5,6,7,7a-tetrahydrobenzofuran-2(4*H*)-one^e,h^	C_11_H_16_O_3_	197.1171	143.16	1.57	0.43	94	81	1.64	0.40	91	89
16	PE(P-16:0e/0:0) ^e,g,h^	C_21_H_44_NO_6_P	438.2977	267.49	1.56	0.44	88	88	–	–	–	–
17	LysoPC(20:3) ^e,g,h^	C_28_H_52_NO_7_P	546.3545	259.45	1.48	0.45	84	81	–	–	–	–
18	Linoleic acid^e,g^	C_18_H_32_O_2_	281.2473	258.56	1.43	2.64	94	94	1.39	2.44	93	93
19	LysoPC(18:3)^e,g,h^	C_26_H_48_NO_7_P	518.3236	237.91	1.33	0.45	78	78	–	–	–	–
20	L-Acetylcarnitine^e,f,g^	C_9_H_17_NO_4_	204.1230	22.91	1.33	2.39	79	84	1.35	2.29	86	80
21	Epsilon-caprolactam^e,f,g,h^	C_6_H_11_NO	114.0914	114.47	1.19	2.08	89	94	1.32	2.58	89	82
22	3-Hexanone^e,g,h^	C_6_H_12_O	101.0960	152.86	–	–	–	–	1.42	0.49	79	93
23	Elaidic acid^e,g^	C_18_H_34_O_2_	283.2629	278.39	–	–	–	–	1.67	3.36	91	98

^a^Experimental monoisotopic mass; ^b^VIP scores derived from OPLS-DA model; ^c^fold change between cancer and control serum calculated from the abundance mean values for each group—cancer-to-normal ratio; ^d^ROC curve analysis for individual biomarkers; ^e^the metabolites identified by high precursor mass accuracy; ^f^the metabolites identified by matching retention time; ^g^the metabolites identified by matching isotopic pattern; ^h^the metabolites identified by matching MS/MS fragment spectra; AUC: area under the curve; CI: confidence interval; FC: fold change; FDR: false discovery rate; HG: high-grade; LG: low-grade; *m/z*: mass-to-charge ratio; RT: retention time; Sens.: Sensitivity; Spec.: Specificity; VIP: variable influence on projection.

### Distinguishing between low- and high- grade bladder cancer and control serum samples

**Figure 2:**
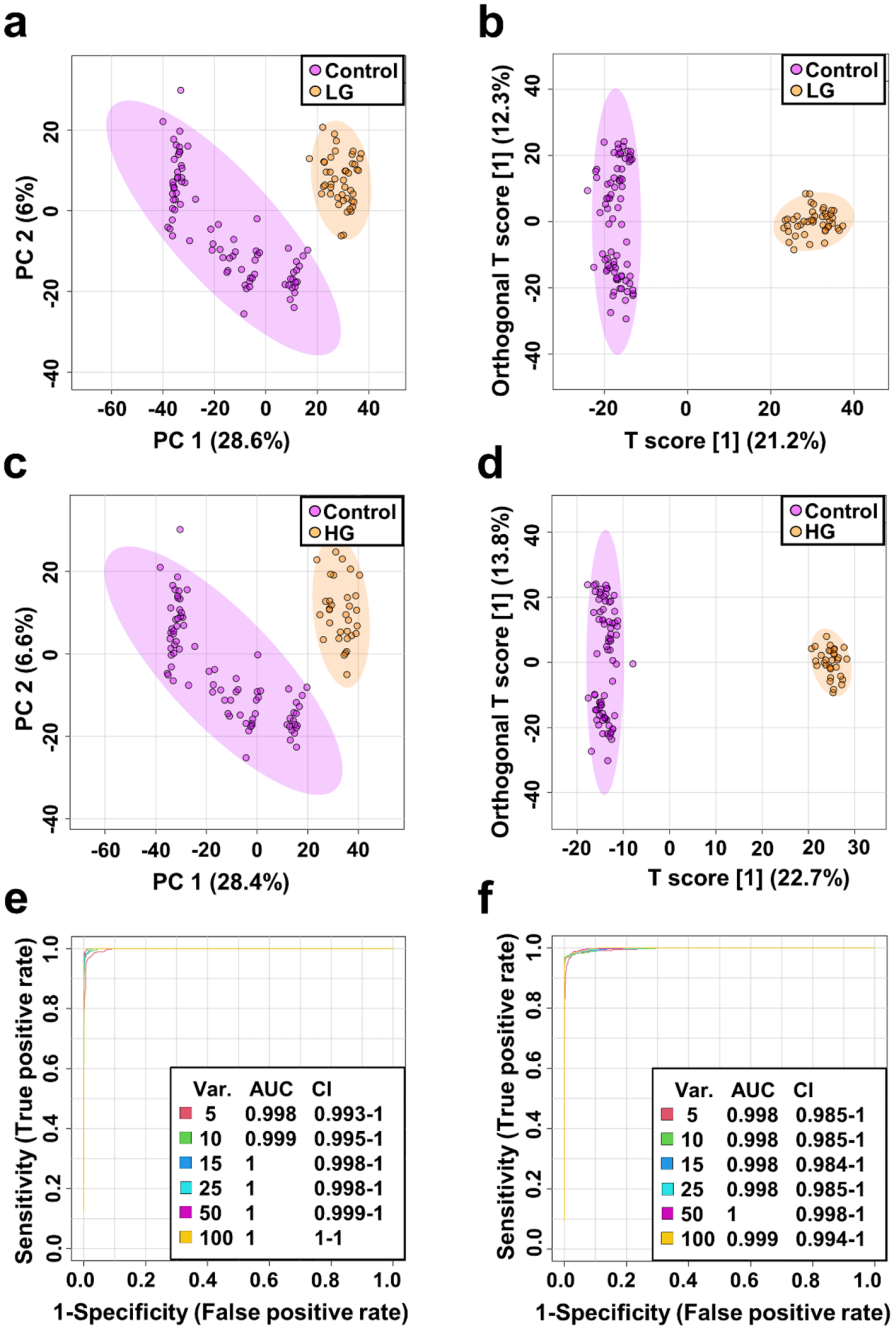
Metabolomic differentiation between different grades of BC and NCs in training set. PCA (**a**) and OPLS-DA (**b**) scores plots of the control (violet) and low-grade (orange). PCA (**c**) and OPLS-DA (**d**) scores plots of the control (violet) and high-grade (orange). ROC curves for LG (**e**) and HG (**f**) BC serum samples vs NCs.

To determine whether metabolomics analysis of serum samples could help discriminate between different grades of BC, another series of PCA and OPLS DA analyses were performed on the training (80 NCs, 32 patients with HG and 45 patients with LG,) and validation (20 NCs, 8 patients with HG and 12 patients with LG,) data sets (Tabe S1) excluding three samples from patients with PUNLMP. PCA and OPLS-DA scores plots revealed good discrimination between separately control and cancer groups of varying grades of tumors (LG vs NCs and HG vs NCs) in both training and validation set (Fig. 2, S2). Quality factors for those models amounted to Q^2^ > 0.89 and R^2^Y > 0.982, with p values based on permutation tests (n = 2000) smaller than 5E-4 (Fig. S3, S4) indicating a perfect discrimination of metabolites profiles between those groups. However, we did not observe a substantial difference between the LG and HG BC patients in the PCA scores plot (data not shown).

In HG BC vs NCs OPLS-DA model 1500 variables were considered as significant (VIP > 1, *p* value < 0.05) in both training and validation set. Among these features, 138 m*/z* values were assigned to a specific chemical compound. Analysis of LG BC vs NCs in OPLS-DA model in training and validation set revealed common 1600 m*/z* values as significant contributors to the separation between those two groups of which 148 were assigned to specific compound. Univariate ROC curve analyses indicated that these models have a good diagnostic performance (Fig. 2, S2). AUC values for five out of fifteen metabolites were found to be greater than 0.75. Finally, set of twenty-three potential LG and HG BC biomarkers were selected with cut-off criteria of FC > 2 and < 0.5, Δ*m/z* < 2 ppm and mSigma < 50 in both testing and validation data sets. The sensitivity and specificity of the selected 23 metabolites were also determined and all metabolites disclosed sensitivity and specificity greater than 78% (Table 2 and S2, Supplementary information 2).

### Distinguishing between different stages of bladder cancer and control serum samples

Analysis of tumor stages was performed for the entire LC–MS dataset of serum metabolite extracts from patients diagnosed with bladder cancer. Metabolite profiling analysis included 69 serum samples from patients with noninvasive papillary carcinomas (pTa) 19 samples from pT1 stage and 12 from patients with muscle invasive bladder cancer (pT2).

**Figure 3:**
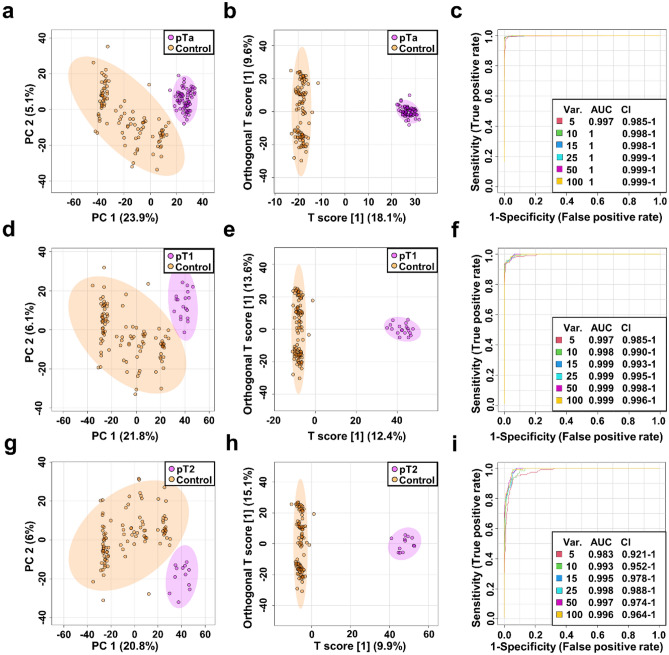
Metabolomic differentiation between different stages of BC and NCs. PCA (**a**), OPLS-DA (**b**) scores plots and ROC curve (**c**) of the pTa (violet) and control (orange). PCA (**d**), OPLS-DA (**e**) scores plots and ROC curve (**f**) of the pT1 (violet) and control (orange). PCA (**g**), OPLS-DA (**h**) scores plots and ROC curve (**i**) of the pT2 (violet) and control (orange).

PCA and OPLS-DA scores plot indicated good separation between NCs and different stages of BC (pTa vs NCs, pT1 vs NCs and pT2 vs NCs, Fig. 3). Quality factors for those models were Q^2^ > 0.904 and R^2^Y > 0.988, with *p* values based on permutation tests (n = 2000) smaller than 5E-4 (Fig. S5) indicating a very good discrimination of metabolites profiles between those groups. Fold Change and VIP plot analysis of the OPLS-DA model indicated 63, 66 and 69 m*/z* values that appeared to be most relevant for sample differentiation between pTa BC vs NCs, pT1 BC vs NCs and pT2 BC vs NCs respectively out of pool of features assigned to specific chemical compounds. Next, ROC curve analysis was performed to assess the performance of three models in distinguishing between pTa; pT1 and pT2 bladder cancer stages and NCs. Finally, set of thirty-seven potential pTa, pT1 and pT2 BC biomarkers were selected with cut-off criteria of FC > 2 and < 0.5, Δ*m/z* < 2 ppm and mSigma < 50 in both testing and validation data sets (Table 3). The sensitivity and specificity of the selected 37 metabolites were also determined and all metabolites disclosed sensitivity and specificity greater than 74 and 62%, respectively S3, Supplementary information 2). Comparison of the three groups of cancer stage (pT1 vs. pTa vs. pT2) did not reveal any statistically significant differences (data not shown).

**Table 3:** Differential metabolites for discrimination between pTa, pT1 and pT2 BC patients and NCs (*p* value < 0.05; FDR < 0.05; VIP > 1; FC < 0.5 and > 2).

No	Metabolites	Formula	*m/z*^a^	RT [s]	pTa versus control		pT1 versus control		pT2 versus control	
					VIP^b^	FC^c^	VIP^b^	FC^c^	VIP^b^	FC^c^
1	Alpha-hydroxyisobutyric acid^d,e^	C_4_H_8_O_3_	87.0439	49.21	–	–	1.20	2.66	1.05	3.92
2	Valeric acid^d,e,f^	C_5_H_10_O_2_	103.0753	132.22	1.90	0.41	1.99	0.41	2.05	0.40
3	Creatinine^d,e,f^	C_4_H_7_N_3_O	114.0661	21.28	–	–	–	–	1.48	2.02
4	Epsilon-caprolactam^d,e,f,g^	C_6_H_11_NO	114.0914	114.47	1.28	2.30	1.51	2.33	1.34	2.46
5	4-Heptanone^d,e,f^	C_7_H_14_O	115.1116	206.71	1.96	0.23	1.99	0.25	2.05	0.23
6	3-Ethylphenol^d,f,g^	C_8_H_10_O	123.0803	122.87	1.88	0.36	1.94	0.35	1.95	0.35
7	D-Limonene^d,f^	C_10_H_16_	137.1324	143.88	1.86	0.30	1.74	0.33	1.87	0.28
8	3,5,5-Trimethyl-2-cyclohexen-1-one^d,f,g^	C_9_H_14_O	139.1116	193.16	2.00	0.17	2.11	0.17	2.17	0.17
9	Thymol^d,e,f^	C_10_H_14_O	151.1116	204.15	1.91	0.39	1.82	0.41	1.84	0.40
10	Perillyl alcohol^d,f^	C_10_H_16_O	153.1273	210.29	2.03	0.25	2.20	0.26	2.31	0.24
11	Methyl 2-octynoate^d,f,g^	C_9_H_14_O_2_	155.1065	177.72	1.89	0.31	1.99	0.32	2.23	0.29
12	1-Acetylindole^d,f,g^	C_10_H_9_NO	160.0757	131.14	1.78	2.09	1.75	2.19	1.70	2.08
13	Umbelliferone^d,f,g^	C_9_H_6_O_3_	163.0389	182.05	1.84	0.49			2.06	0.46
14	1-Phenyl-1-pentanone^d,f,g^	C_11_H_14_O	163.1116	200.50	1.94	0.33	2.04	0.36	2.04	0.37
15	4,7-Dimethyl-1,3-benzothiazol-2-ylamine^d,f,g^	C_9_H_10_N_2_S	179.0638	142.04	–	–	1.82	2.02	–	–
16	Benzophenone^d,f,g^	C_13_H_10_O	183.0809	226.43	1.56	2.00	1.45	2.14	–	–
17	L-Acetylcarnitine^d,e,f^	C_9_H_17_NO_4_	204.1230	22.91	1.46	2.15	1.48	2.37	1.53	2.81
18	Aureonitol^d,f,g^	C_13_H_18_O_2_	207.1378	173.22	1.97	0.20	2.00	0.22	2.11	0.20
19	4,4,7a-trimethyl-3a,5,6,7-tetrahydro-3*H*-indene-1-carboxylic acid^d,f,g^	C_13_H_20_O_2_	209.1534	197.75	1.89	0.40	1.87	0.42	1.99	0.38
20	7-Epi-Jasmonic acid^d,f,g^	C_12_H_18_O_3_	211.1328	160.86	1.97	0.19	2.03	0.20	1.99	0.21
21	Cys-Pro^d,f,g^	C_8_H_14_N_2_O_3_S	219.0797	91.87	1.03	2.34	–	–	–	–
22	Pro-Leu^d,f,g^	C_11_H_20_N_2_O_3_	229.1546	46.53	–	–	–	–	1.14	2.35
23	Alantolactone^d,f,g^	C_15_H_20_O_2_	233.1535	194.63	1.95	0.23	1.99	0.25	1.94	0.27
24	Curcumol^d,f,g^	C_15_H_24_O_2_	237.1848	226.69	1.12	0.23				
25	Isovalerylcarnitine^d,e,f^	C_12_H_23_NO_4_	246.1697	121.55	1.16	2.18	1.11	2.49	1.07	2.45
26	Linoleic acid^d,f^	C_18_H_32_O_2_	281.2473	258.56	1.59	2.35	1.53	2.62	1.49	2.73
27	Elaidic acid^d,f^	C_18_H_34_O_2_	283.2629	278.39	1.87	3.35	2.02	3.86	1.73	2.91
28	PE(P-16:0e/0:0)^d,f,g^	C_21_H_44_NO_6_P	438.2977	267.49	1.59	0.49	1.95	0.39	2.08	0.36
29	Cefazolin^d,f,g^	C_14_H_14_N_8_O_4_S_3_	455.0371	135.59	–	–	1.02	288.76	1.30	62.93
30	LysoPE(P-18:0/0:0)^d,f,g^	C_23_H_48_NO_6_P	466.3288	294.22	1.72	0.39	1.90	0.34	2.16	0.28
31	LysoPC(14:0/0:0)^d,f,g^	C_22_H_46_NO_7_P	468.3080	236.23	–	–	–	–	1.96	0.33
32	LysoPC(P-18:0)^d,g^	C_26_H_54_NO_6_P	508.3756	294.32	–	–	–	–	1.60	0.48
33	LysoPC(18:2)^d,f,g^	C_26_H_50_NO_7_P	520.3393	246.39	–	–	–	–	1.80	0.44
34	LysoPC(20:3)^d,f,g^	C_28_H_52_NO_7_P	546.3545	259.45	–	–	1.57	0.45	1.76	0.39
35	LysoPC(22:5)^d,f,g^	C_30_H_52_NO_7_P	570.3546	255.47	–	–	–	–	1.48	0.45
36	PC(16:1/16:1)^d,g^	C_40_H_76_NO_8_P	730.5380	312.51	–	–	1.61	0.15	–	–
37	PC(16:0/18:3)^d,g^	C_42_H_78_NO_8_P	756.5535	313.85	–	–	1.19	0.42	–	–

^a^ Experimental monoisotopic mass; ^b^VIP scores derived from OPLS-DA model; ^c^fold change between cancer and control serum calculated from the abundance mean values for each group—cancer-to-normal ratio; ^d^the metabolites identified by high precursor mass accuracy; ^e^the metabolites identified by matching retention time; ^f^the metabolites identified by matching isotopic pattern; ^g^the metabolites identified by matching MS/MS fragment spectra; AUC: area under the curve; FC: fold change; FDR: false discovery rate; *m/z*: mass-to-charge ratio; pT1 and pTa—high risk non-muscle invasive bladder cancer; pT2—muscle invasive bladder cancer; RT: retention time; VIP: variable influence on projection.

### Pathway analysis of potential biomarkers

**Figure 4:**
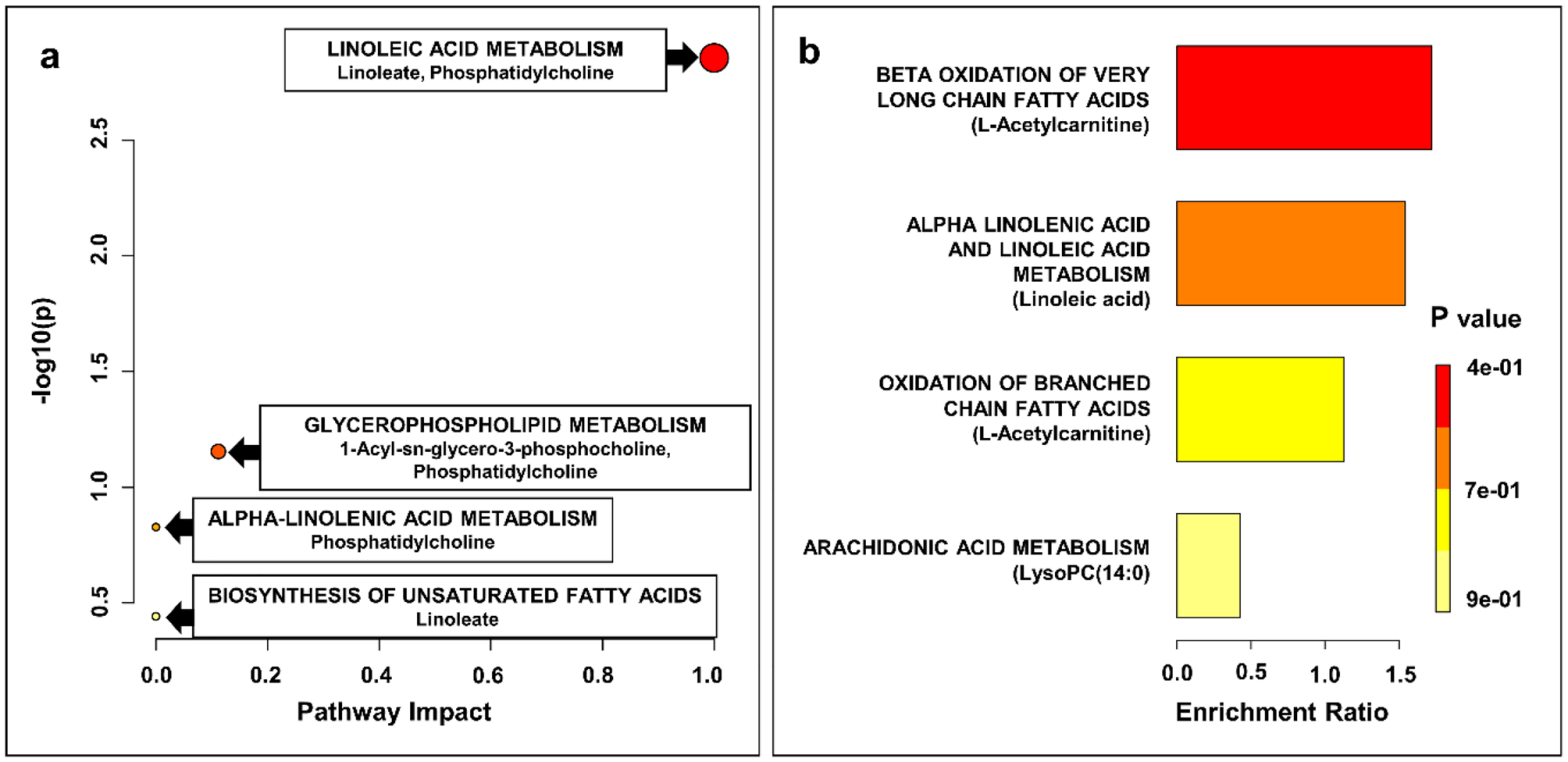
Results of pathway topology analysis of selected statistically significant metabolites in BC. A Pathway analysis based on KEGG (**a**); bubble area donating to the impact of each pathway with color representing the significance from highest in red to lowest in white; (**b**) Quantitative enrichment analysis based on SMPDB.

A metabolic pathway impact analysis was performed using MetaboAnalyst 5.0 to identify the most relevant pathways involved in the observed changes of serum metabolite levels. Forty-five metabolites identified in the UHPLC-UHRMS analysis were subjected to pathway analysis and quantitative pathway enrichment analysis. Forty-nine compounds were found to be relevant to human metabolism. Five metabolic pathways, including linoleic acid metabolism, glycerophospholipid metabolism, alpha-linolenic acid metabolism, arachidonic acid metabolism and biosynthesis of unsaturated fatty acids were found to be significantly impacted comparing BC to controls. Results from pathway impact analysis is shown in Fig. 4a and Table S2 (supplementary information 1).

To expand the metabolomic analysis of pathways related to bladder cancer, we performed a quantitative enrichment analysis using the MetaboAnalyst 5.0 pathway enrichment module and its associated Small Molecule Pathway Database (SMPDB). Two additional pathways were found to be relevant to bladder cancer: beta oxidation of very long chain fatty acids, phospholipid biosynthesis and oxidation of branched chain fatty acids (Fig. 4b and Table S3.

## Discussion

Over the past decade, metabolomics studies have provided valuable information on the metabolic profile of patients suffering from various diseases, including cancer, and identified potential markers of developing or recurring disease. Cancer cells have the ability to reprogram their metabolism in order to support the increased need for energy caused by rapid proliferation. Monitoring of changes in the levels of various metabolites in cancer cells or body fluids may be a potential source of new cancer biomarkers. To date, many studies have been published indicating the high potential of metabolomic markers in the diagnosis of various cancers and in understanding of the mechanisms of cancer initiation and development^28^.

In this study UHPLC-UHRMS and -UHRMS/MS methods were employed to evaluate changes in serum metabolite levels between 100 bladder cancer patients and 100 normal controls. The largest class of compounds differentiating the NCs group from the BC patients were lipids and lipid-like molecules. Lipids are the fundamental building blocks of all cell membranes and serve as a long-term energy storage. Furthermore, lipids have many other important functions within living organisms including transmit nerve impulses, production and regulation of certain hormones, cushion vital organs, intracellular signal transmission and cell transporting systems. Lipid metabolism is involved in various processes associated with cancer cells. Over the past decade, numerous studies have demonstrated that lipids and metabolites associated with lipid metabolism may be potential markers in human cancers including bladder cancer^29^. We found that the plasma content of 10 glycerophospholipids including PE(P-16:0e/0:0), PC(16:1/16:1), PC(16:0/18:3), LPE(P-18:0/0:0), LPC(14:0/0:0), LPC(P-18:0), LPC(18:3), LPC(18:2), LPC(20:3), LPC(22:5) were significantly higher in the serum of NCs than in the BC subjects. This finding is in line with previous metabolomic studies that demonstrated an association of changes in the levels of these lipids in the blood with various cancers^30^. Thus, alterations in these lipids’ metabolism may, therefore, play important roles in the development and progression of bladder cancer.

Glycerophospholipids (GPs), also called phospholipids include phosphatidylethanolamines (PE), phosphatidylcholines (PC) and phosphatidylethanolamines (PE), all of which are glycerol-based phospholipids. These compounds are a major component of the membranes of animal cells in which they are asymmetrically distributed acting as the matrix of different membrane proteins. Many previous studies have found low serum PE levels in various cancers including colon, prostate, lung, and breast cancers indicating these compounds as potential tumor markers^31,32^. Serum levels of PE(P-16:0e/0:0) were found by Lin et. al significantly lower in patients with kidney cancer compared to controls^22^. Some studies have provided evidence that translocation of PE from the inner to the outer leaflet of the plasma membrane indicating a loss of asymmetric distribution of aminophospholipids has been shown as the first sign of impending apoptosis. Thus, lower levels of PE(P-16:0e/0:0) in serum may be an early symptom of apoptotic cell death^33^. Moreover, human phosphatidylethanolamine-binding protein is associated with resistance to apoptosis of tumor cells^34^. It has been reported that exogenous PEs inhibits the growth and indicates an apoptosis of human hepatoma HepG2 cells^35^. Lysophosphatidylethanolamine LPC, LysoPC), LysoPE (P-18:0/0:0) also known as LPE(18:0) was found in lower level in plasma of patients with liver, gastric colorectal, ovarian and lung cancer compared to the control group^32,36,37^. Lysophosphatidylcholines (LPC, lysoPC) are an important endogenous signaling phospholipids involved in a variety of important processes, including cell migration, cell proliferation, inflammation and angiogenesis. Decreased LPC plasma level in cancer was also observed in previous study and was associated with body weight loss and increased inflammation. Level of these compounds is inversely correlated with C-reactive protein levels in plasma (CRP)^38^. LPCs were found to be disturbed in several diseases including cancer. Previous metabolomic studies have reported lower level of PC(34:4), LysoPC(20:3) and LPC(P-18:0) in plasma of patients with ovarian cancer (EOC) compared to control^39^. Zhang et al.^40^ have reported that LPC(14:0) was down-regulated in patients with recurrent EOC. Lower level of lysophospholipids have been associated with high activity of specific cell-surface G protein-coupled receptors which may cause apoptosis. Tan et al.^41^ observed significantly lower of LPC(14:0) in the serum of patients with colorectal cancer compared with healthy controls. LPC(18:1), LPC(18:2) and LPC(18:3) were significantly decreasing in plasma of patients with colorectal cancer compared with healthy controls^42,43^. Lee et al.^32^ showed that the levels of LPC(18:2) were lower in plasma samples of patients with colorectal cancer and higher in plasma samples of patients with liver, gastric, lung and thyroid compared to those of healthy control individuals using UHPLC-MS/MS. LPC(18:2) was also found in lower level in plasma of patients with ovarian cancer compared to the control group^36^. Previous metabolomic studies have demonstrated that LysoPC(18:1), LysoPC(20:3) were down-regulated in patients with ovarian cancer. Four of these compounds including LPC(14:0), LPC(18:3), LPC(20:3), LPC(22:5) were previously related to kidney injury. Metabolic profiling of plasma from patients with cancer cachexia revealed significantly lower levels of LPC(14:0), LPC(P-18:0), LPC(18:2), LPC(20:3), LPC(22:5) and LPE(18:0) compared to healthy controls^44^. Three of these six LPC including LPC(18:1), LPC(18:3), LPC(22:5) we identified previously at lower levels in serum of patients with thyroid carcinoma^45^. To our knowledge, only one lipid out of the ten most differentiating both groups cancer and control we indicated has been previously associated with bladder cancer. Tan et al.^18^ indicated slightly higher level of LPC(18:2) in serum of patients with BC compared to controls using UHPLC-Q-ToF MS.

Lower levels of four prenol lipids including perillyl alcohol, D-limonene, thymol, alantolactone were found in serum of BC compared to controls. These monoterpenoids commonly occurring in many plants are known for their anti-tumor, antioxidant, anti-inflammatory and anti-fungal activity. Thymol and limonene have been shown to inhibit bladder cancer cell proliferation and induces these cells apoptosis^46,47^.

We found that serum levels of metabolites: L-acetylcarnitine, linoleic acid and elaidic acid were higher and three others: valeric acid and 7-epi-jasmonic acid lower in BC patients compared to NCs. The levels of linoleic and elaidic acid were also found as significantly higher in patients with colorectal cancer^48^. Increased serum activity of acetylcarnitine have been previously pointed out as a potential tumor biomarkers^49,50^. Acetylcarnitine is a naturally occurring acetic acid ester of carnitine, important in mitochondrial tricarboxylic acid (TCA) cycle activity. Increased urine levels of this compound have previously been reported in patients with BC^51^. Elevation of acetylcarnitine may be an indication of decreased carbon flow into the TCA cycle or excess production of acetyl-CoA^52^. Previous studies revealed elevated urine level of acetylcarnitine and isovalerylcarnitine in BC patients compared to controls^53,54^. However, the association between isovalerylcarnitine and bladder cancer has not yet been explained.

In order to apply the correct treatment regimens for BC patients, in addition to indicating the neoplasm, it is necessary to precisely and accurately indicate the stage and grade of this cancer. In total, 23 differential metabolites were identified as potential marker for discriminating between LG and HG BC patients and NCs. Among these metabolites, 18 metabolites were the common characteristic of both LG and HG BC patients. Three metabolites including lysoPC(20:3), PE(P-16:0e/0:0) and 2(4H)-benzofuranone, 6-Hydroxy-4,4,7a-trimethyl-5,6,7,7a-tetrahydrobenzofuran-2(4*H*)-one were identified in much higher level only in the serum of patients with HG BC, while four metabolites including 3-hexanone, diethylene glycol 2-ethylhexyl ether, elaidic acid, umbelliferone were found in significant higher level only in the serum of patients with LG BC patients.

In total, 38 differential metabolites were identified as potential marker for discriminating between pTa, pT1 and pT2 BC patients and NCs. Among these metabolites, 22 metabolites were the common to all three stages of BC. Two metabolites including Cys-Pro and curcumol were identified in much higher levels only in the serum of patients with pTa BC, while two metabolites including LysoPC(20:3) and alpha-hydroxyisobutyric acid were found in significant higher level only in the serum of patients with pT1 BC patients. Moreover, five metabolites including norcamphor, creatinine, dihydrojasmone, pro-leu, palmitoleoyl ethanolamide were found in significant higher level only in the serum of patients with pT2 BC patients.

We demonstrate that ultra-high-resolution mass spectrometry is a powerful tool for the characterization of the serum metabolome differences in BC. Twenty-seven potentially robust metabolic biomarkers were identified for 100 tumor serum samples from patients with BC patients after comparison against 100 healthy controls owing to the excellent predictive ability of AUC > 0.99. We also identified twenty-three metabolites that might be used as potential biomarkers to distinguish LG and HG and thirty-seven metabolites that may serve to differentiate between the pTa/pT1 and pT2 stages of BC. Our results suggest that differential serum metabolite profiles and can help identify patients with BC compared with NCs, with significant discriminating power between different stages and grades of bladder cancer. Our findings, may potentially provide facile and less invasive diagnostic methodology for detection of different stages and grades of bladder cancer and recurrent disease management. In the future, a new class of biomarkers of BC could contribute to development of non-invasive, highly specific and sensitive diagnostic tests that could be employed to aid the detection of new tumors and also predict recurrences.

## Supplementary Information

Supplementary Information 1.

Supplementary Information 2.

## Data Availability

The data that support the findings of this study is available from the corresponding author upon reasonable request.
