# Supplementary material for: Untargeted ultra-high-resolution mass spectrometry metabolomic profiling of blood serum in bladder cancer
Source: Sci Rep. 2022 Sep 7;12:15156. doi: 10.1038/s41598-022-19576-9 (PMC9452537; doi:10.1038/s41598-022-19576-9)
Supplement: Supplementary file 1 — Supplementary Information 1. [file 41598_2022_19576_MOESM1_ESM.docx]

**Untargeted ultrahigh-resolution mass spectrometry metabolomic profiling of blood serum in bladder cancer**

Joanna Nizioł^a*^, Krzysztof Ossoliński^b^, Artur Kołodziej^c^, Aneta Płaza-Altamer^c^, Anna Ossolińska^b^, Tadeusz Ossoliński^b^, Tomasz Ruman^a^

*^a^Rzeszów University of Technology, Faculty of Chemistry, 6 Powstańców Warszawy Ave., 35-959 Rzeszów, Poland, e-mail: jniziol@prz.edu.pl*

*^b^Department of Urology, John Paul II Hospital, Grunwaldzka 4 St., 36-100 Kolbuszowa, Poland*

*^c^Doctoral School of Engineering and Technical Sciences at the Rzeszów University of Technology, 8 Powstańców Warszawy Ave., 35-959, Rzeszów, Poland*

*Corresponding author: Joanna Nizioł, e-mail: jniziol@prz.edu.pl, tel: (+48 17) 865-1896

**Keywords:** Human serum, bladder cancer, biomarkers, metallomics, metabolomics, LC-MS

**Table of contents**

**Table S1.** Clinical characteristic of bladder cancer patients **2**

**Figure S1.** OPLS-DA model validation for distinction of bladder cancer and control samples **3**

**Figure S2.** Metabolomic analysis of serum samples from HG/LG BC and NCs in the validation set. **4**

**Figure S3.** OPLS-DA model validation for distinction of LG BC and NCs. **5**

**Figure S4.** OPLS-DA model validation for distinction of HG BC and NCs**6**

**Figure S5.** OPLS-DA model validation for distinction of stages of BC and NCs. **7**

**Table S2.** Result from Pathway Analysis **8**

**Table S3.** Result from Enrichment Pathway Analysis **9**

**Table S1.** Summary of clinical characteristics of bladder cancer patients

| **Characteristics** | **Training set (n = 80)** | | **Validation set (n= 20)** | |
| --- | --- | --- | --- | --- |
|  | **Bladder cancer patients** | **Normal controls** | **Bladder cancer patients** | **Normal controls** |
| **No. of subjects** | 80 | 80 | 20 | 20 |
| **Age (mean/SD)** | 73/10 | 74/10 | 75/8 | 64/12 |
| **Sex** |  |  |  |  |
| Male | 64 | 62 | 17 | 15 |
| Female | 16 | 18 | 3 | 5 |
| **Grade^a^** |  |  |  |  |
| High grade | 32 | - | 8 | - |
| Low grade | 45 | - | 12 | - |
| PUNLMP | 3 | - | 0 | - |
| **Stage** |  |  |  |  |
| pTa | 55 | - | 14 | - |
| pT1 | 15 | - | 4 | - |
| pT2 | 10 | - | 2 | - |

*^a^* Tumors were classified according to World Health Organization (WHO)/ International Society of Urological Pathology (ISUP) classification criteria. LG – Low-grade; HG – high-grade; PUNLMP - papillary urothelial neoplasm of low malignant potential; pT1 and pTa – high risk non-muscle invasive bladder cancer;

pT2 – muscle invasive bladder cancer; pT- the stage has been based on pathological or microscopic findings;

**Figure S1.** OPLS-DA model validation of LC-MS data for distinction of bladder cancer and control samples. (A) The permutation test showing the observed and cross-validated R^2^Y and Q^2^ coefficients based on 2000 permutations of LC-MS data from two groups of bladder serum samples with a statistically significant p value < 5E-4 (0/2000) in the training set (A) and validation set (C). Model overview showing the R^2^X, R^2^Y and Q^2^ coefficients for the groups in the training set (B) and validation set (D).

**Figure S2.** Metabolomic analysis of serum samples from HG/LG BC and NCs in the validation set. PCA and OPLS-DA scores plots of the tumor (violet) and control (orange) serum samples in the training set (Figure 1A and C) and validation set (Figure 1B and D). The receiving operator characteristic (ROC) curves in the LG BC (E) and HG BC (F).

**Figure S3.** OPLS-DA model validation of LC-MS data for distinction of LG BC and NCs. (A) The permutation test showing the observed and cross-validated R^2^Y and Q^2^ coefficients based on 2000 permutations of LC-MS data from two groups of bladder serum samples with a statistically significant p value < 5E-4 (0/2000) in the training set (A) and validation set (C). Model overview showing the R^2^X, R^2^Y and Q^2^ coefficients for the groups in the training set (B) and validation set (D).

**Figure S4.** OPLS-DA model validation of LC-MS data for distinction of HG BC and NCs. (A) The permutation test showing the observed and cross-validated R^2^Y and Q^2^ coefficients based on 2000 permutations of LC-MS data from two groups of bladder serum samples with a statistically significant p value < 5E-4 (0/2000) in the training set (A) and validation set (C). Model overview showing the R^2^X, R^2^Y and Q^2^ coefficients for the groups in the training set (B) and validation set (D).

**Figure S5.** OPLS-DA model validation of LC-MS data for distinction of different stages of BC and NCs. (A) The permutation test showing the observed and cross-validated R^2^Y and Q^2^ coefficients based on 2000 permutations of LC-MS data from two groups of bladder serum samples with a statistically significant p value < 5E-4 (0/2000). Model overview showing the R^2^X, R^2^Y and Q^2^ coefficients for the groups in the training set.

**Table S2.** Result from Pathway Analysis

| **KEGG pathway** | **Total**^a^ | **Hits**^b^ | **Impact**^c^ | ***P-*value**^d^ | | **Holm p**^e^ | | **FDR**^f^ | |
| --- | --- | --- | --- | --- | --- | --- | --- | --- | --- |
| Linoleic acid metabolism | 5 | 2 | 1.000 | 1.4E-03 | 0.117 | | 1.2E-01 | |  |
| Glycerophospholipid metabolism | 36 | 2 | 0.112 | 7.0E-02 | 1.000 | | 1.0E+00 | |  |
| alpha-Linolenic acid metabolism | 13 | 1 | 0.000 | 1.5E-01 | 1.000 | | 1.0E+00 | |  |
| Arachidonic acid metabolism | 36 | 1 | 0.000 | 3.6E-01 | 1.000 | | 1.0E+00 | |  |
| Biosynthesis of unsaturated fatty acids | 36 | 1 | 0.000 | 3.6E-01 | 1.000 | | 1.0E+00 | |  |

^a^The total number of compounds in the pathway; ^b^the hits is the actually matched number from the NMR and data; ^c^the pathway impact value calculated from pathway topology analysis; ^d^*P-*value calculated from the enrichment analysis; ^e^*P-*value adjusted by Holm–Bonferroni method; ^f^*P-*value adjusted using False Discovery Rate; KEGG: Kyoto Encyclopedia of Genes and Genomes

**Table S3.** Result from Enrichment Pathway Analysis

| **SMPDB pathway** | **Total**^a^ | **Hits**^b^ | **Expected**^c^ | ***P-*value**^d^ | **Holm p**^e^ | **FDR**^f^ |
| --- | --- | --- | --- | --- | --- | --- |
| Beta Oxidation of Very Long Chain Fatty Acids | 17 | 1 | 0.581 | 4.5E-01 | 1 | 1 |
| Alpha Linolenic Acid and Linoleic Acid Metabolism | 19 | 1 | 0.649 | 4.9E-01 | 1 | 1 |
| Oxidation of Branched Chain Fatty Acids | 26 | 1 | 0.889 | 6.0E-01 | 1 | 1 |
| Arachidonic Acid Metabolism | 69 | 1 | 2.360 | 9.2E-01 | 1 | 1 |

^a^The total number of compounds in the pathway; ^b^the hits is the actually matched number from the NMR data; ^c^the pathway impact value calculated from pathway topology analysis; ^d^*P-*value calculated from the enrichment analysis; ^e^*P-*value adjusted by Holm–Bonferroni method; ^f^*P-*value adjusted using False Discovery Rate; SMPDB: The Small Molecule Pathway Database
